# *Fusobacterium nucleatum* Alters Atherosclerosis Risk Factors and Enhances Inflammatory Markers with an Atheroprotective Immune Response in ApoE^null^ Mice

**DOI:** 10.1371/journal.pone.0129795

**Published:** 2015-06-16

**Authors:** Irina M. Velsko, Sasanka S. Chukkapalli, Mercedes. F. Rivera-Kweh, Hao Chen, Donghang Zheng, Indraneel Bhattacharyya, Pandu R. Gangula, Alexandra R. Lucas, Lakshmyya Kesavalu

**Affiliations:** 1 Department of Periodontology, College of Dentistry, University of Florida, Gainesville, Florida, United States of America; 2 Department of Oral Biology, College of Dentistry, University of Florida, Gainesville, Florida, United States of America; 3 Cardiovascular Medicine and Molecular Genetics & Microbiology, College of Medicine, University of Florida, Gainesville, Florida, United States of America; 4 Oral Diagnostic Sciences, College of Dentistry, University of Florida, Gainesville, Florida, United States of America; 5 Department of Oral Biology and Research, CWHR Meharry Medical College, Nashville, Tennessee, United States of America; 6 Department of Physiology, CWHR Meharry Medical College, Nashville, Tennessee, United States of America; The Forsyth Institute, UNITED STATES

## Abstract

The American Heart Association supports an association between periodontal disease (PD) and atherosclerotic vascular disease (ASVD) but does not as of yet support a causal relationship. Recently, we have shown that major periodontal pathogens *Porphyromonas gingivalis* and *Treponema denticola* are causally associated with acceleration of aortic atherosclerosis in ApoE^null^ hyperlipidemic mice. The aim of this study was to determine if oral infection with another significant periodontal pathogen *Fusobacterium nucleatum* can accelerate aortic inflammation and atherosclerosis in the aortic artery of ApoE^null^ mice. ApoE^null^ mice (n = 23) were orally infected with *F*. *nucleatum* ATCC 49256 and euthanized at 12 and 24 weeks. Periodontal disease assessments including *F*. *nucleatum* oral colonization, gingival inflammation, immune response, intrabony defects, and alveolar bone resorption were evaluated. Systemic organs were evaluated for infection, aortic sections were examined for atherosclerosis, and inflammatory markers were measured. Chronic oral infection established *F*. *nucleatum* colonization in the oral cavity, induced significant humoral IgG (*P*=0.0001) and IgM (*P*=0.001) antibody response (12 and 24 weeks), and resulted in significant (*P*=0.0001) alveolar bone resorption and intrabony defects. *F*. *nucleatum* genomic DNA was detected in systemic organs (heart, aorta, liver, kidney, lung) indicating bacteremia. Aortic atherosclerotic plaque area was measured and showed a local inflammatory infiltrate revealed the presence of F4/80^+^ macrophages and CD3^+^ T cells. Vascular inflammation was detected by enhanced systemic cytokines (CD30L, IL-4, IL-12), oxidized LDL and serum amyloid A, as well as altered serum lipid profile (cholesterol, triglycerides, chylomicrons, VLDL, LDL, HDL), in infected mice and altered aortic gene expression in infected mice. Despite evidence for systemic infection in several organs and modulation of known atherosclerosis risk factors, aortic atherosclerotic lesions were significantly reduced after *F*. *nucleatum* infection suggesting a potential protective function for this member of the oral microbiota.

## Introduction

Atherosclerotic vascular diseases (ASVD) are the leading cause of death globally [1]. Although there are numerous well-established factors that increase risk for ASVD, including genetic factors, hypertension, hypercholesterolemia and smoking, these do not account for all cases, and microbial infection also is now considered an important risk factor for ASVD [1]. Periodontal disease (PD) causing infectious oral microbes are commonly detected in atherosclerotic plaques. Periodontal disease is characterized by a chronic polymicrobial biofilm that induces inflammatory response in gingival tissues surrounding and supporting the teeth. The inflamed periodontal tissues permit entry of over 275 oral bacterial species into the blood stream through damaged capillaries during chewing, flossing, tooth brushing, and dental procedures [2,3]. Although the American Heart Association supports an association between PD and ASVD, a meta-analysis of currently available data has determined that there is not as yet sufficient evidence to support a definitive causal relationship [1]. We are systematically investigating individual periodontal disease pathogens that have been linked to ASVD by a physiologically relevant model of chronic oral infection in hyperlipidemic ApoE^null^ mice. Our published studies demonstrated significantly increased atherosclerosis in ApoE^null^ mice after oral *P*. *gingivalis* [4], and *T*. *denticola* [5], as well as polybacterial infections [6].

*Fusobacterium nucleatum* is a periodontal pathogen that is implicated in development of several systemic diseases such as atherosclerosis [7], Alzheimer’s disease [8], colorectal cancer [9–12], and adverse pregnancy outcomes [13], and is the oral pathogen most commonly found at sites of systemic infection [14]. *F*. *nucleatum* is one of the most abundant species found in the periodontal pocket [14], and levels of *F*. *nucleatum* are elevated at sites of periodontal disease [14,15]. Within the oral cavity, *F*. *nucleatum* is an intermediate colonizer in the subgingival biofilm and aggregates together with numerous other oral species. These co-aggregates are thought to aid in development of the oral biofilm [16]. In particular, *F*. *nucleatum* may help attachment of late colonizers *P*. *gingivalis*, *T*. *denticola*, and *Tannerella forsythia* which are strongly implicated in PD development [17]. *P*. *gingivalis* and *T*. *denticola* are rarely found in periodontal pockets without *F*. *nucleatum* [16], highlighting a potential critical role in disease. In support of this, both subcutaneous co-infection studies and oral co-infection studies have demonstrated that inclusion of *F*. *nucleatum* synergistically enhances bacterial virulence and disease severity [18–20]. Most *in vivo* studies examining the association between PD and ASVD have used *P*. *gingivalis*, which is one of the best studied periodonto-pathogenic bacteria; however *P*. *gingivalis* is not the only oral bacteria associated with atherosclerotic lesion progression [5–7,21], nor is the only one found in atherosclerotic plaques [22–26].

*F*. *nucleatum* is associated with more systemic diseases than other known periodontal pathogens, and has been isolated from more than 10 sites of systemic infection [14], and infection with *F*. *nucleatum* has been linked to development of colon cancer [9–12], adverse pregnancy outcomes [27,28], arterial atherosclerotic plaque growth [7], as well as dementia and brain abscess [14]. The exact role that PD and periodontal pathogens, specifically *F*. *nucleatum*, play in the development of atherosclerosis is, however, as yet unclear. *In vitro* and *in vivo* studies have shown that heat-killed *F*. *nucleatum* and the *F*. *nucleatum* heat-shock protein GroEL are capable of inducing foam cell formation in cultured human monocytic THP-1 cells *in vitro*, as well as significant growth of atherosclerotic plaque in ApoE^null^ mice after intravenous inoculation with either whole cells or recombinant GroEL [7], yet this model cannot draw conclusions concerning the role of PD and periodontal-derived bacteria in atherogenesis. Due to the fact that gingival infection with *F*. *nucleatum* and subsequent atherosclerosis development has not been examined to date this study is critical in elucidating a clear association between the pathogen and disease.

This study examined the invasive capacity of *F*. *nucleatum* in initiation and progression of atherosclerosis using a chronic gingival infection model of ApoE^null^ mice. We report here that *F*. *nucleatum* colonizes the mouse oral cavity, spreads hematogenously, and modulates the local aortic immune response, but nevertheless *F*. *nucleatum* does not elicit a strong inflammatory response or significant atherosclerotic lesion development, but rather orchestrates systemic changes in the host leading to an unexpected late reduction in plaque progression.

## Materials and Methods

### Bacterial Growth Conditions, Mouse Strain and Oral Infection

**Fig 1:**
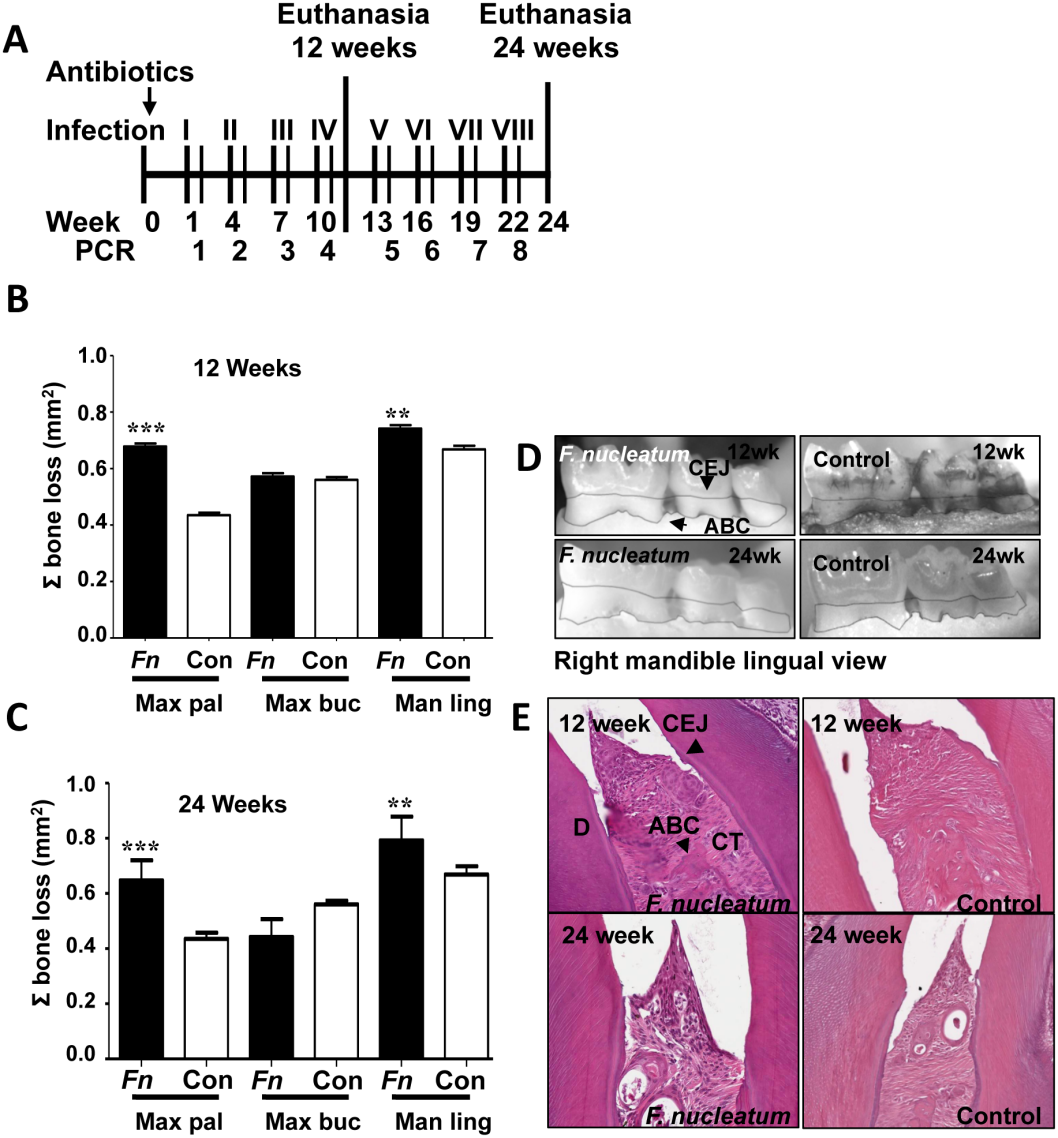
Oral infection schedule and periodontal disease parameters. (A) Oral infection schedule. (B) Twelve-week infection-induced horizontal alveolar bone resorption was statistically significant on the maxilla palatal and mandible lingual sides. (C) Twenty-four-week infection-induced horizontal ABR was statistically significant on the maxilla palatal, maxilla buccal, and mandible lingual sides. (D) Representative left maxilla lingual view images of horizontal ABR measurements, outlining the area between the cemento-enamel junction (CEJ) and alveolar bone crest (ABC) of molars 1, 2, and 3 of 12- and 24 week *F*. *nucleatum*-infected and sham-infected mice. (E) Representative images of gingival histology of 12- and 24 week-infected and sham-infected mice demonstrating minimal gingival inflammation. * *P*<0.05, ** *P*<0.01, *** *P*<0.001.

*F*. *nucleatum* ATCC 49256 was grown on blood agar plates in a Coy anaerobic chamber at 37°C for 2 days. Cells were suspended in reduced transport fluid (RTF]–4% carboxymethylcellulose (CMC) for oral infection [29]. Male ApoE^null^ B6,129P2-Apoetm1Unc/J mice, 8 weeks-old were ordered from the Jackson Laboratories (Bar Harbor, ME). Mice were acclimated and infected by oral lavage as described [6] in a chronic infection model (Fig 1A). Twenty-four mice were randomly assigned to the infection group, and 24 mice to the sham-infected group. Control mice were mock infected with sterile vehicle (RTF-4% CMC). Gingival plaque samples were taken after three days of each infection cycle (8 samples) with a sterile veterinary cotton swab, and suspended in 150μl TE (Tris-EDTA) buffer (Fig 1A). Cardiac blood was collected during euthanasia at 12 and 24 weeks and sera were stored at -20°C for immunoglobulin G (IgG) and IgM antibody analysis. Jaws of 9 mice were removed, for evaluation of alveolar bone resorption (ABR) by morphometric analysis. Jaws from 3 mice were suspended in 10% buffered formalin and decalcified for histology. All mice procedures were approved by University of Florida Institutional Animal Care and Use Committee (IACUC, protocol #201004539).

### Detection of *F*. *nucleatum* Genomic DNA

Gingival plaque samples were suspended in TE buffer, and then used directly as template in the PCR mix. Aorta, heart, liver, spleen, lungs, and kidney were homogenized with a QIAGEN TissueRuptor and genomic DNA extracted using the QIAGEN DNEasy Blood and Tissue kit (QIAGEN, Valencia, CA) [4,5,6]. PCR was performed on both oral samples and systemic organ samples with the following 16S rDNA *F*. *nucleatum* specific primers (Integrated DNA Technologies): forward 5’- TAAAGCGCGTCTAGGTGGTT -3’, reverse 5’- AC**A**GCTTTGC**G**ACTCTCTGT -3’ [6,23]. Bold underlined letters were altered from the reference for a 100% match with strain ATCC 49256.

### Analysis of Alveolar Bone Resorption

The mandibles and maxillae of nine mice in each group were collected for histomorphometric analysis as previously described [6]. Both horizontal alveolar bone resorption and intrabony defects were determined as previously described [6]. Measurements represent the average ABR determined by three independent viewers blinded to the groups.

### Gingival Inflammation

The mandibles and maxillae of three mice from *F*. *nucleatum*-infected and sham-infected mice were fixed in 10% neutral buffered formalin, decalcified with Immunocal (Decal Chemical Corporation, Tallman, NY) for 7 days. The decalcified tissue blocks were embedded in paraffin and sections were prepared at 5 μm followed by staining with hematoxylin and eosin [6]. The stained slides were scanned at 20x using an APerio Scan Scope, and histological measurements were taken using APerio ImageScope v11.0.2.725 software [30]. Inflammation was quantified by measuring the distance of gingival apical epithelial migration and counting the number of infiltrating leukocytes per 50μm^2^ area of gingival tissue, by a reviewer blinded to the groups.

### Serum Antibody Analysis

*F*. *nucleatum*-specific serum IgG and IgM antibody titers were determined by ELISA, using a standard ELISA protocol as described previously [31]. Mean antibody titer values of infected mice were divided by mean antibody titer values of sham-infected mice, and the quotient represents the mean fold change in *F*. *nucleatum*-specific antibody titer due to infection. Graphs show mean fold-change in *F*. *nucleatum*-specific antibody titer of infected mice. Statistically significant differences between mean *F*. *nucleatum*-specific antibody titers of infected and sham-infected mice were determined by two-tailed Student’s t test, and are indicated above fold-change values on the graph.

### *In vivo* Localization of *F*. *nucleatum* by Fluorescence *in situ* Hybridization (FISH)

FISH was used to detect metabolically active bacteria [4,5,32]. FISH was performed on formalin-fixed paraffin embedded jaw and aorta tissue sections. *F*. *nucleatum* 16S rRNA-specific oligonucleotide FUSO 5’-CTAATGGGACGCAAAGCTCTC-3’[33] labeled with Alexa fluor-568 was ordered from Invitrogen (Carlsbad, CA). The protocol was performed as previously described [4]. Slides were viewed at 63X with a Leica DMIRB microscope equipped with a Photometrics cascade-cooled EMCCD camera, controlled by the open-source software package μManager (http://www.micro-manager.org/). Images were processed using Image J (NCBI).

### Atherosclerotic Plaque Analysis

Aortic arch (n = 6) and thoracic aortas (n = 6) were fixed in 10% neutral buffered formalin and embedded in paraffin. Sectioning and hematoxylin and eosin staining were performed as previously described [5]. Plaque area, intimal layer thickness, medial layer thicknesses were measured and intimal/medial layer thickness ratios were calculated using ImagePro software by a blinded reviewer. Intimal/medial thickness ratios are used to reduce variations produced by differing vessel sizes [4–6].

### Quantification of Aortic Inflammatory Cell Infiltration

Sections measured for atherosclerotic plaque were subsequently selected and stained for analysis of inflammatory cell infiltration. Each aortic section examined for atherosclerotic plaque (n = 6 infected and control) was again evaluated for presence of CD3^+^ T cells and F4/80^+^ macrophages by immunohistochemical staining, as described previously [4].

### Serum Lipid Profile and SAA Level

Blood was collected at euthanasia, and sera were separated by centrifugation. Thirty microliters of 24-week-infected mice (n = 6) and sham-infected (n = 6) mouse sera were analyzed by gel filtration high performance liquid chromatography (HPLC) analysis for lipid profiles [cholesterol, triglycerides, chylomicrons, very low-density lipoprotein (VLDL), low-density lipoprotein (LDL), high-density lipoprotein (HDL), (Skylight Biotech Inc, Akita, Japan) as described previously [4,5]. Levels of serum amyloid A (SAA) of 24-week-infected mice (n = 6) and sham-infected mice (n = 6) were measured using a prepared ELISA kit from Kamiya Biomedical (Seattle, WA) [4,5].

### Serum Nitric Oxide (NO) Measurement

Serum NO (μM) concentrations were measured using a nitric oxide fluorometric assay kit (Bio Vision Inc., Milpitas, CA) from 24-week-infected (n = 6) and sham-infected (n = 6) mice [4–5].

### RT^2^ Profiler PCR Array

Expression of 84 genes known to be involved in pathogenesis of atherosclerosis was examined in aortas of infected (n = 3) and control (n = 3) mice by qPCR with the RT^2^ Profiler Mouse Atherosclerosis PCR Array (SA Biosciences) [4,5]. Tissues were homogenized by QIAGEN TissueRuptor (QIAGEN, Valencia, CA). RNA was extracted using an RNeasy kit (QIAGEN, Valencia, CA), followed by reverse transcription with the RT^2^ First Strand Kit (QIAGEN, Valencia, CA). Samples were prepared for array with the SYBR Green Master mix (QIAGEN, Valencia, CA). Cycling was performed following manufacturer’s protocol, and data was analyzed using the manufacturer’s PCR Array Data Analysis V4 excel worksheet [4,5].

### RayBio Mouse Inflammatory Cytokine Array

Sera from 12-week-infected and sham-infected (n = 10) and 24 week-infected and sham-infected (n = 10) mice were pooled and used to analyze 40 different cytokines on the Ray Biotech Mouse Inflammatory Cytokine Array, as per manufacturer’s protocol [4,5]. Array slides were read with a GenePix 4400 scanner, using GenePix Pro 7.2.29.002 software. Results were analyzed using the RayBio Analysis Tool excel sheet [4,5].

### Statistical analysis

Statistical analyses of ELISA, serum lipid profile, SAA, NO and horizontal alveolar bone resorption were performed using an unpaired two-tailed Student’s t test, with GraphPad Prism software v.5 [5,6]. *P* values less than 0.05 were considered statistically significant. ELISA, horizontal alveolar bone resorption, SAA and NO graphs show mean with standard deviation. Aortic histology and immunohistochemistry measurements were analyzed by ANOVA with the Statview program and *post hoc* PLSD analysis, and graphs are represented as mean with standard error.

## Results

### Oral Colonization and Periodontal Disease Induction

*F*. *nucleatum* genomic DNA was detected in the oral cavity of 19 out of 24 mice by the first infection (Table 1) and all mice tested positive for *F*. *nucleatum* after at least one infection during the infection period. It is possible that the sampling technique was not sensitive enough to detect subgingival *F*. *nucleatum*, which may explain why not all mice had consistently positive samples.

**Table 1:** Detection of oral microbial plaque samples for *F*. *nucleatum* genomic DNA.

	Total mice/Oral Sample #							
	n = 24				n = 12			
Group	1	2	3	4	5	6	7	8
*F*. *nucleatum*	19	9	7	8	3	1	0	4
Control	0	0	0	0	0	0	N/D	N/D

Oral samples were assessed by PCR as described in the methods. 0—PCR was performed and no samples were positive for *F*. *nucleatum* DNA. N/D—not done: no plaque samples were taken to allow undisrupted bacterial growth.

Twelve week-infected mice had statistically significant alveolar bone resorption relative to control mice in the maxilla palatal (0.68 v. 0.43 mm^2^) (*P* = 0.0001) and mandible lingual sides of the jaw (0.74 v. 0.66 mm^2^) (*P* = 0.002) (Fig 1B and 1D). Twenty-four week-infected mice developed highly significant bone resorption relative to control mice in the maxilla palatal (0.65 v. 0.43 mm^2^) (*P* = 0.0001) and mandible lingual sides (0.80 v. 0.66 mm^2^) (*P* = 0.003) (Fig 1C and 1D) of the jaw. Intrabony defects (vertical resorption away from the tooth root) were observed in 20% of 12 weeks infected mice versus 9% of controls, and in 13% of 24 weeks infected mice versus 5% in controls. *F*. *nucleatum*-induced bone resorption was comparable or similar to *P*. *gingivalis* and *T*. *denticola*-induced alveolar bone resorption and intrabony defects [4,5].

Histological analysis of jaw sections revealed minimal inflammation and epithelial hyperplasia in infected ApoE^null^ mice at both 12 and 24 weeks (Fig 1E). Apical migration of junctional epithelium was not observed in infected or sham-infected mice at either time points, nor was the number of lymphocytes in the gingival tissues different between infected and control mice at either 12 or 24 weeks of infection. Viable *F*. *nucleatum* was not detected by FISH within gingival tissues of 12 or 24 weeks infected ApoE^null^ mice.

### *F*. *nucleatum* elicits a Significant Humoral Antibody Response and Disseminates Systemically

**Fig 2:**
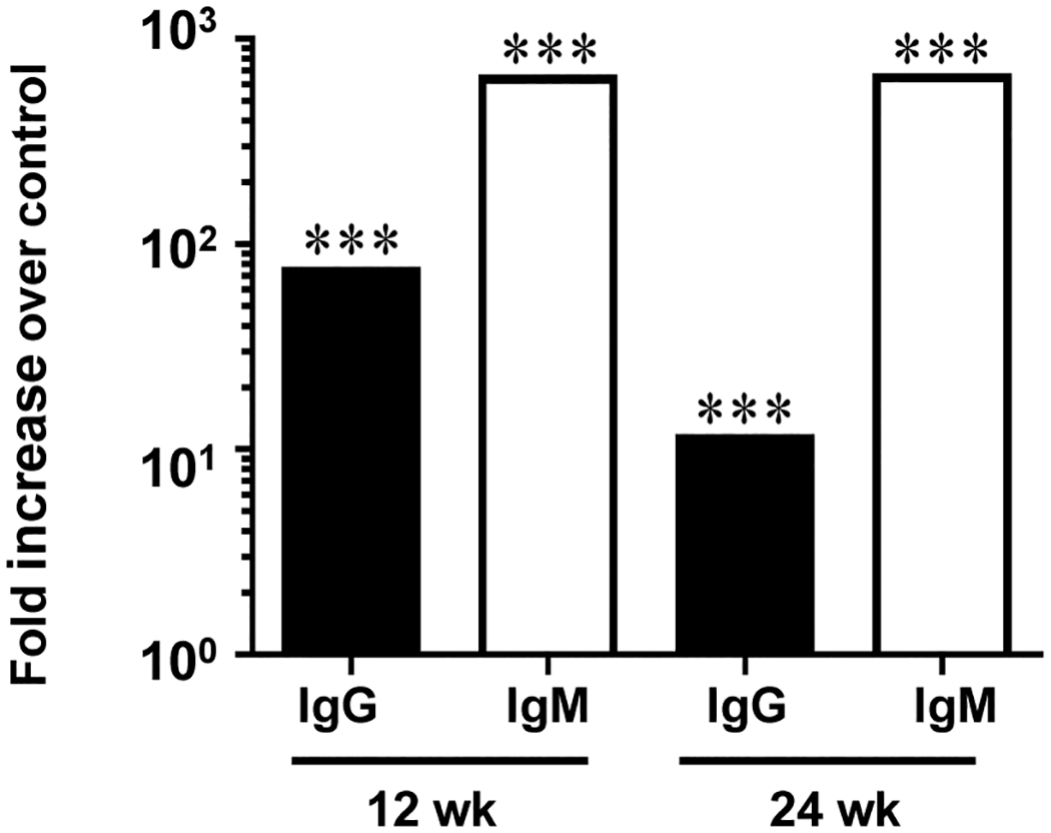
Chronic oral infection with *F*. *nucleatum* induced significant levels of serum *F*. *nucleatum*-specific antibodies. Graphs represent the fold-increase in *F*. *nucleatum*-specific IgG or IgM antibody titer in infected mice over control mice at both 12 and 24 weeks of infection. (*** *P*<0.001).

Serum antibody response to periodontal pathogens is further evidence of bacterial infection. ELISA antibody analysis of serum samples from 12 week-infected mice showed significantly higher IgG levels in infected mice (approximately 70-fold) than control mice (Fig 2, *P* = 0.0001). Similarly, IgM antibody levels in 12 week-infected mice were significantly greater (approximately 700-fold) than control mice (*P* = 0.0001). In addition, IgG levels of 24 week-infected mice were significantly higher (approximately 15-fold) than controls (*P* = 0.0001), as were IgM levels of 24 week-infected mice (approximately 700-fold) than controls (*P* = 0.001) (Fig 2).

To determine if bacteria spread systemically from the mouse gingival tissue, DNA was extracted from mouse heart, aorta, liver, spleen, kidney and lungs at both 12 and 24 weeks of infection, and *F*. *nucleatum* specific PCR was used to detect *F*. *nucleatum* genomic DNA [34, 5, 6]. In 12-week infected mice, *F*. *nucleatum* DNA was detected in 10 out of 12 hearts, and 5 out of 6 aortas, 6 out of 12 livers, 3 out of 12 kidneys, and 7 out of 12 lungs (Table 2). In 24-week infected mice, *F*. *nucleatum* genomic DNA was detected in 5 out of 12 hearts, 6 out of 6 aortas, 2 out of 12 kidneys, 1 out of 12 lungs, (Table 2). These data clearly indicate that *F*. *nucleatum* spread hematogenously from gingival connective tissues to systemic organs.

**Table 2:** Detection of *F*. *nucleatum* genomic DNA in internal organs.

			Positive systemic tissue samples					
			Number of organs with *F*. *nucleatum* DNA					
Weeks of infection	Group	# of mice	Heart	Aorta*	Liver	Spleen	Kidney	Lung
12	*F*. *nucleatum*	12	10	5	6	0	3	7
	Control	12	0	0	0	0	0	0
24	*F*. *nucleatum*	12	5	6	0	0	2	1
	Control	12	0	0	0	0	0	0

*F*. *nucleatum* genomic DNA was detected by PCR as described in the methods.

* N = 6.

### Chronic Oral Infection Induces Minimal Atherosclerotic Plaque at 24 weeks

**Fig 3:**
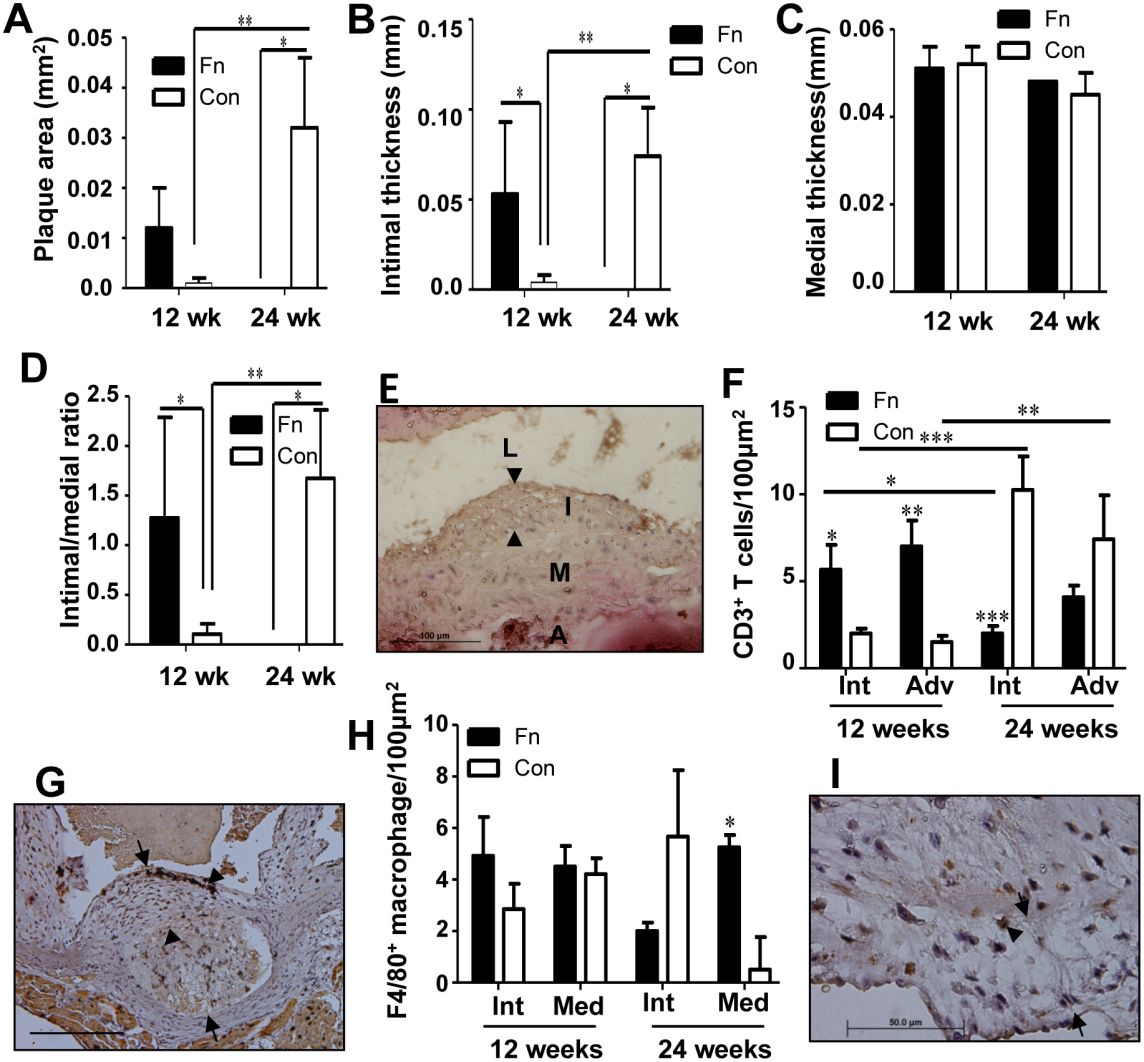
Chronic oral infection results in an unexpected reduction in plaque area at 24 weeks. (A) Infected mouse plaque area was significantly reduced when compared to sham-infected mice at 24 weeks of infection. Control mouse plaque area was significantly increased at 24 weeks relative to 12 weeks. (B) Intimal thickness at 12 and 24 weeks. (C) Medial thickness at 12 and 24 weeks. (D) Intimal/medial thickness ratios again indicate a significant reduction in plaque at 24 weeks in infected mice when compared to controls. (E) Twenty-four week-infected mouse aorta demonstrating plaque. Scale bar is 100μm. Arrowheads indicate plaque. (F) CD3^+^ T cell counts were significantly higher in 12 week-infected mice than controls and also higher than 24 week-infected mice. (G) Twelve-week infected mouse aorta stained for CD3^+^ T cells. Arrows define plaque margins, arrow heads point to CD3^+^ stained cells (brown staining). Scale bar is 100μm. (h) F4/80^+^ macrophage counts in 12 week-infected mice were unaffected. (I) Twenty-four week-infected mouse aorta stained for F4/80^+^ cells. Arrows define plaque margins, arrow heads point to F4/80^+^ stained cells. Scale bar is 50μm. Fn—*F*. *nucleatum*, Con—control, L—lumen, I, Int—intimal layer, M—medial layer, A, Adv—adventitial layer. * *P*<0.05, ** *P*<0.01, *** *P*<0.001.

Twelve week- and 24 week-infected mice did not develop significant aortic plaque (Fig 3A and 3E). In fact, less plaque was detected in 24 week-infected mice than 12-week-infected mice (Fig 3A). Twenty-four week-infected mice developed minimal aortic plaque, which was significantly smaller than sham-infected mice (*P* = 0.014) (Fig 3A), while control mice developed significantly larger plaques at 24 weeks compared to 12-week controls (*P* = 0.001). The intimal thickness of infected mice was significantly greater than controls at 12 weeks (Fig 3B, *P* = 0.032), while it was significantly less than controls at 24 weeks (*P* = 0.015). Similar to plaque area, control mice exhibited significantly greater intimal thickness at 24 weeks than at 12 weeks (*P* = 0.002). Medial thickness was unaffected by infection at either time points (Fig 3C). The intimal/medial layer thickness ratio, which is used to normalize measurements of plaque size to variable arterial sizes, of 12-week-infected mice was significantly greater than in controls (*P* = 0.036). However, as for the plaque areas, this ratio of intimal-to-medial thickness was reversed by 24 weeks (*P* = 0.015), while control mice exhibited significantly greater intimal/medial ratios at 24 weeks compared to 12 weeks (*P* = 0.004) (Fig 3D). These data indicate that chronic oral infection with *F*. *nucleatum* as monoinfection alone does not promote atherosclerosis induction, and may conversely inhibit plaque formation. *F*. *nucleatum* were not detected by FISH within aortic tissues of infected mice at either 12 or 24 weeks of infection.

Immunohistochemical staining of aortic sections revealed significantly greater numbers of CD3^+^ T cells in infected than control mice at 12 weeks of infection in the intimal (*P* = 0.02) and adventitial layers (*P* = 0.033) of the aorta (Fig 3F and 3G). By 24 weeks of infection, these cell numbers had reversed, and the number of T cells in the intima were significantly less than in control mice (*P* = 0.0001), and the intimal T cell infiltration were significantly less in 24-week-infected mice than in 12 week-infected mice (*P* = 0.036). The number of T cells in control mouse intimal and adventitial layers was significantly greater at 24 weeks than at 12 weeks (*P* = 0.0001, *P* = 0.001, respectively). There was no significant difference in the number of F4/80^+^ macrophages detected in the intimal or medial layers of 12-week-infected mice relative to controls (Fig 3H and 3I). However, 24-week-infected mice had significantly elevated numbers of F4/80^+^ cell in the media (*P* = 0.026) versus controls. Together a lack of increased infiltration of macrophages in infected mice and a reduction in infiltration of T cells into the intimal and adventitial layers at 24 weeks would suggest that chronic *F*. *nucleatum* infection reduces inflammation in the aorta, and may explain in part the minimal atherosclerotic plaque development.

### Oral Infection and Systemic Dissemination Promote Atherosclerosis-permissive Conditions

**Fig 4:**
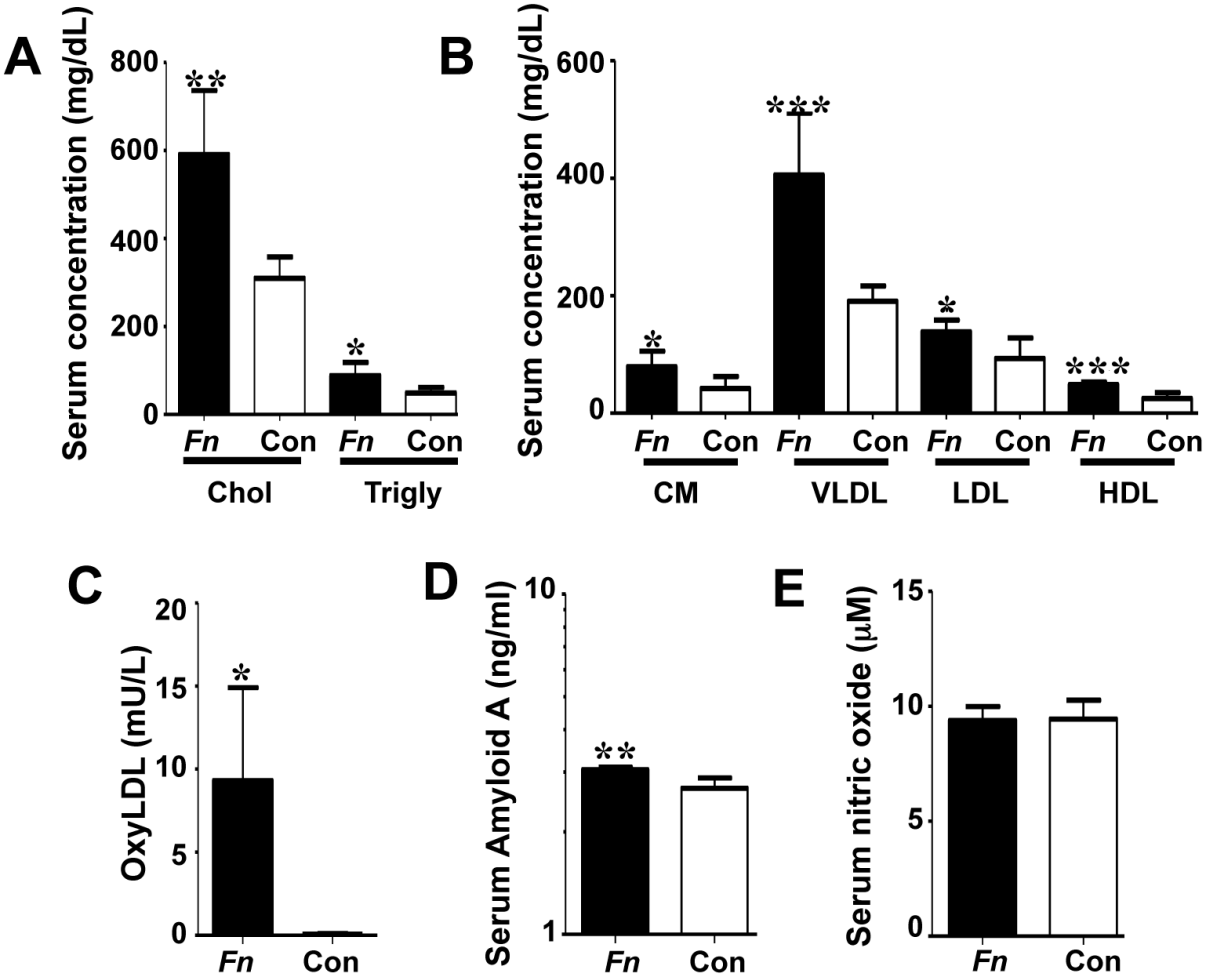
Chronic oral infection with *F*. *nucleatum* significantly alters serum risk factors for atherosclerosis. (A) Twenty four-week-infected mice (n = 6) developed significantly elevated levels of total serum cholesterol and total serum triglycerides relative to sham-infected mice (n = 6). (B) *F*. *nucleatum* infected mice developed significantly elevated levels of chylomicrons (CM), very low density lipoproteins (VLDL), low density lipoprotein (LDL) and high density lipoproteins (HDL) relative to sham-infected mice (n = 6). (C) Infection significantly increased the total serum oxyLDL in infected mice. (D) Infected mice exhibited significantly elevated acute phase inflammatory protein serum amyloid A at 24 weeks. (E) Infection with *F*. *nucleatum* did not significantly alter the concentration of serum nitric oxide at 24 weeks of infection. *Fn*—*F*. *nucleatum* infected mice, Con—control, Chol—cholesterol, Trigly—triglycerides, CM—chylomicrons, VLDL—very low density lipoprotein, LDL—low density lipoprotein, HDL—high density lipoprotein. * *P*<0.05, ** *P*<0.01, *** *P*<0.001.

Serum lipoprotein profiles were significantly altered in *F*. *nucleatum*-infected mice relative to controls at 24 weeks of infection (Fig 4). Total serum cholesterol and triglycerides were significantly elevated with *F*. *nucleatum* infection (*P* = 0.001and *P* = 0.015, respectively) (Fig 4A). Serum lipoprotein fractions from 24 weeks infected mice were also significantly elevated relative to controls, including chylomicrons (CM) (*P* = 0.022), very low-density lipoprotein (VLDL) (*P* = 0.001), low-density lipoprotein (LDL) (*P* = 0.019), and high-density lipoprotein (HDL) (*P* = 0.0003) (Fig 4B). Additionally, the alterations in lipid levels indicate increased levels of both protective HDL and potentially pro-atherogenic LDL and VLDL, suggesting a possible balancing of effects by *F*. *nucleatum* on lipid levels. Serum oxyLDL, a risk factor for plaque development, was significantly elevated (Fig 4C, *P* = 0.0001) in 24 weeks infected mice relative to controls. Twenty-four week-infected mice had significantly elevated levels of SAA relative to controls (Fig 4D, *P* = 0.002), indicating an inflammatory hepatic response to acute infection and elevated systemic inflammatory burden. Serum NO, an indicator of vascular endothelial function, was not altered in infected mice at 24 weeks relative to controls (Fig 4E), suggesting no vascular endothelial dysfunction, consistent with the minimal plaque observed in the infected mice.

### Aortic Gene Expression Changes Indicate Modified Vascular Inflammation

In 24-week-infected mice aortic tissue, expression of 12 genes was increased by greater than 2-fold (Table 3). These included the Th2 response genes *Csf2*, *Il1r2*, *Il3*, *Il4*, and *Il5*. This enhanced Th2 response would suggest decreased inflammation in the 24 week-infected mice. Additionally, the anti-apoptotic regulator *Birc3* was up-regulated, cell surface molecules E-selectin (*Sele*) and vascular cell adhesion molecule 1 (*Vcam1*) were up-regulated, and coagulation regulators serine protease inhibitor B1 (*Serpinb1*, leukocyte elastase inhibitor) and E1 (*Serpine1*, plasminogen activator inhibitor-1 or PAI-1) were up-regulated. In some prior mouse models of vascular injury PAI-1 has been reported as protective against inflammatory cell activation and invasion.

**Table 3:** Atherosclerosis-related gene expression changes during 24 week infection.

Gene grouping	Gene	Fold change	P-value
Apoptosis	*Birc3*	2.08	>0.05
	*Bcl2*	-2.85	>0.05
Blood clotting/ coagulation cascade	*Serpinb2*	4.49	>0.05
	*Serpine1*	2.30	>0.05
	*Npy*	-6.54	>0.05
	*Fga*	-10.6	>0.05
	*Fgb*	-8.04	>0.05
Immune response	*Csf2*	4.07	>0.05
	*Il1a*	4.79	>0.05
	*Il1r2*	8.57	>0.05
	*Il3*	2.73	>0.05
	*Il4*	2.44	>0.05
	*Il5*	4.00	>0.05
	*Klf2*	-2.03	>0.05
	*Spp1*	2.02	>0.05
	*Tgfb2*	-2.14	>0.05
Leukocyte/endothelial cell adhesion	*Itga5*	-2.07	>0.05
	*Mmp3*	-2.42	>0.05
	*Sele*	2.15	>0.05
	*Thbs4*	-2.90	0.01
	*Vcam1*	2.14	>0.05
	*Cd44*	-4.89	>0.05
	*Col3a1*	-7.59	>0.05
	*Eln*	-6.05	>0.05
Lipid transport/ metabolism	*ApoA1*	-7.06	>0.05
	*ApoB*	-3.16	>0.05
	*Msr1*	-2.37	>0.05

N = 3. Fold change represents increase or decrease in expression relative to sham-infected mice.

Fifteen genes were down-regulated more than 2-fold (Table 3). These included lipoproteins *Apoa1* and *Apob*, transcriptional regulator *Klf2* and the macrophage scavenger receptor *Msr1*; matrix components *Col3a1*, *Eln*, *Fga*, *Fgb*, *Mmp3*, and *Tgfb2*; cell adhesion molecules *Cd44*, *Itga5*, and *Thbs4*; apoptotic regulator *Bcl2*; and neuropeptide Y (*Npy*), which contributes to macrophage formation, platelet aggregation and vascular smooth muscle cell proliferation [35]. ApoB, matrix metalloproteases, adhesion molecules and growth factors such as TGFβ are also associated with elevated in inflammatory atheroma development suggesting these reductions may be protective.

### Serum Inflammatory Cytokine Levels Indicate Suppressed Inflammation during Chronic Infection

The number of elevated serum cytokines in infected mice was greater at 12 weeks than at 24 weeks of infection (Table 4). Eighteen cytokines were elevated >2-fold relative to controls at 12 weeks of infection, including CD 30L, Fas ligand, Fractalkine, GCSF, IFN-γ, IL1-β, IL4, IL10, IL12p40/p70, IL12p70, IL17, I-TAC, Lymphotactin, MCP-1, MCSF, MIG, MIP-1α, and TIMP-2. Four cytokines were decreased relative to controls at 12 weeks: BLC, Eotaxin, Eotaxin-2, and LIX. At 24 weeks of infection, fewer cytokines than at 12 weeks were altered 2-fold relative to controls. These cytokines elevated more than 2-fold was CD-30L, IL1-β, IL4, IL13, and Lymphotactin. Eight cytokines were decreased >2-fold at 24 weeks, including BLC, Eotaxin, Eotaxin-2, Fas ligand, IL6, LIX, MCP-1, and sTNF RI (Table 4). Reductions in Eotaxin-2 and MCP-1, which are chemoattractants for resting and memory T cells, respectively, correlate with the reduced numbers of T cells detected in the aortic vessel wall at 24 weeks. Additionally, the return of inflammatory mediators IFN-γ, IL1-β, and IL12p40/p70 in infected mice to levels comparable to sham-infected mice further supports a reduction of inflammation during chronic infection.

**Table 4:** Serum cytokine changes in 24 weeks *F*. *nucleatum*-infected mice.

	12 week infection		24 week infection	
Cytokine grouping	Cytokine	Fold change	Cytokine	Fold change
Cell activation and proliferation	CD30L	270	CD30L	4.1
	GCSF	3.9	IL-1β	3.2
	IFN-γ	2.6	IL4	51
	IL-1β	2.3	IL6	-2.2
	IL4	181	IL13	10
	IL10	2.6	sTNF RI	-2.7
	IL-12p40/p70	1920		
	IL-12p70	2.2		
	IL-17	-3.2		
Leukocyte chemoattractants	BLC	-5.8	BLC	-2.1
	Eotaxin	-4.6	Eotaxin	-2.6
	Eotaxin-2	-5.7	Eotaxin-2	-3.2
	LIX	-15	LIX	-5.6
	MCP-1	2.1	MCP-1	-2.4
	MIP-1α	3.6		
T cell chemoattractants	Fractalkine	2	Lymphotactin	4.1
	ITAC	2.1		
	Lymphotactin	2		
	MCSF	2		
	MIG	2.1		
ECM molecules	TIMP-2	2.3		
Apoptosis	Fas ligand	5.8	Fas ligand	-6.1

N = 10. Pooled samples for infected and control groups. Both pooled samples were run in duplicate and all concentrations averaged. Fold change represents increase or decrease in average expression relative to sham-infected controls. Only cytokines altered 2-fold or greater from controls of the equivalent time point are included.

## Discussion

Genomic DNA of nine periodontal pathogens has been detected in atherosclerotic plaques by PCR, yet the majority of *in vivo* studies on the relationship between infection with periodontal pathogens and development of atherosclerotic plaque have focused on the well-characterized pathogen *P*. *gingivalis* [4,36,37]. Given the polymicrobial nature of dental plaque, it is important to assess the atherogenic potential of other well-characterized oral pathogens with significant PD associations. In support of this, fusobacterial genomic DNA has been detected in atherosclerotic cardiovascular specimens by PCR [26,38] and *F*. *nucleatum* is closely associated with other PD infections, suggesting a contribution to development of atherosclerosis, yet whether in a pro-atherogenic or protective capacity is unclear. Frequent involvement of *F*. *nucleatum* in extra-oral systemic infections [14], and colon cancer [10–12], supports a role for this species in atherogenesis, as the bacteria are able to survive, hematogenously spread, and replicate at sites distant from the oral cavity. In support of this, the *F*. *nucleatum* heat shock protein GroEL was shown to promote atherosclerotic lesion development in ApoE^null^ mice [7], albeit under conditions of high-fat diet. Here, we demonstrate that chronic oral infection with *F*. *nucleatum* induces PD symptoms in ApoE^null^ mice, and that *F*. *nucleatum* is able to spread by hematogenous routes and to modulate host immune response and atherosclerotic risk factors, with evidence for both pro- and anti-inflammatory responses and yet does not promote atherosclerotic lesion progression.

Previous *in vitro* studies showed that *F*. *nucleatum* is a potent B cell-mitogen [39], for which we observe strong evidence in the significantly elevated levels of IgG and IgM during chronic infection of mice. The mitogenic activity is attributed to the outer membrane porin FomA, which is a TLR2 adjuvant [40]. Some of the systemic antibodies to *F*. *nucleatum* may provide a protective response, as we detected genomic DNA of *F*. *nucleatum* by PCR in fewer systemic organs of 24 week-infected mice than 12-week-infected mice.

Oral infection with *F*. *nucleatum* significantly altered the serum lipid profile levels. There was evidence for a trend toward a more pro-atherogenic state, by elevating total cholesterol and triglycerides, VLDL and LDL, but also the HDL fraction, which is considered more protective against atheroma formation. HDL is a key molecule with anti-atherogenic properties due to its broad range of functions like reverse cholesterol transport as well as decreasing inflammation, inhibition of thrombosis, stimulation of fibrinolysis, and modulation of immune cells involved in atherosclerosis, especially monocyte-macrophages, B and T lymphocytes [41,42]. Interestingly, although all of the lipid fractions examined were statistically elevated in *F*. *nucleatum*-infected mice, atherosclerotic lesions did not develop. This is in contrast to our observation in *P*. *gingivalis*-infected ApoE^null^ mice, which did not exhibit statistically significantly elevated serum lipoprotein particle fractions, yet developed significant atherosclerotic lesions [4], and *T*. *denticola*-infected ApoE^null^ mice, which exhibited significantly elevated cholesterol and VLDL and developed significant plaque lesions [5]. It is possible that the significant increase in serum HDL observed with oral *F*. *nucleatum* infection is sufficient to counter the effects of hyperlipidemia, so that the lipid shift, while apparently pro-atherogenic, has a net zero effect on the mouse vasculature.

Additionally, our previous monoinfection studies with *P*. *gingivalis* [4] or *T*. *denticola* [5] significantly elevated the serum oxyLDL, a particular risk factor for atherosclerosis, yet the data presented here demonstrate elevated oxyLDL alone to be insufficient to induce significant atherosclerotic plaque growth. These apparently contradictory findings demonstrate differences in the ability of different bacterial species to induce atherosclerotic plaque development. Notable difference between *F*. *nucleatum* monoinfection and the recently published *P*. *gingivalis* and *T*. *denticola* monoinfection studies is that serum NO was not significantly decreased in *F*. *nucleatum*-infected mice as it was in the *P*. *gingivalis* and *T*. *denticola* monoinfections [4,5]. Decreased serum NO is an indicator of endothelial dysfunction, which is thought to contribute to atherosclerotic lesion initiation [43]. As both the *P*. *gingivalis* and *T*. *denticola* monoinfected mice demonstrated significant atherosclerotic plaque accumulation, while *F*. *nucleatum* monoinfected mice did not, we hypothesize that bacterial-induced endothelial dysfunction may be an important mediator of infection-induced atherosclerotic plaque development.

*F*. *nucleatum* uses its FadA adhesion to bind to and invade both endothelial cells and epithelial cells [44,45], giving it a high potential for active roles in inducing PD and atherosclerosis. Binding of FadA to vascular endothelial cadherin was shown to mediate internalization of *F*. *nucleatum* into HUVECs by loosening cell-cell junctions and increasing permeability [45]. Given this ability, it is unexpected that we detected no bacteria within gingival tissues or aortic tissues by FISH. It is possible that shallower or deeper cuts may have presented invasive *F*. *nucleatum*, or that invasion frequency is too low to be detected, or that *F*. *nucleatum* does not readily infect vascular tissues, or this may be due to differences in mouse and human physiology, as *F*. *nucleatum* is adapted to survive in humans, but is not known to be part of the mouse flora. An example of this phenomenon was demonstrated by Guo, *et al*. [46], who found that the *F*. *nucleatum* outer membrane porin FomA is able to bind the Fc of human IgG but not of mouse IgG.

Our assessment of aortic gene expression changes revealed similarities in the response to infection by different periodontal bacterial species. There was much overlap in gene expression changes between *F*. *nucleatum*-infected mice and the published *P*. *gingivalis*- and *T*. *denticola*-infected mice [4,5], indicating the local immune response to infection by different bacterial species to be similar. However, mice infected with *F*. *nucleatum* had more extracellular matrix and cell adhesion molecules down-regulated than either previous studies at 24 weeks, including *CD44*, *Col3a1a*, *Eln*, *Itga5*, *Klf2*, *Mmp3* and *Thbs4*. This may inhibit significant inflammatory cell infiltration of the aortic vessel and thereby reduce the inflammatory processes that contribute to atherosclerotic plaque development, and is a possible explanation for the significantly reduced numbers of T cells detected throughout the aortic vessel at 24 weeks of infection relative to 12 weeks. Additionally, there appears to be reduced T cell recruitment at 24 weeks, as the infected mice exhibit reduced serum T cell chemoattractants Eotaxin-2 and MCP-1.

Serum changes in the relative amounts of FasL in infected mice were drastic, reducing from a 6-fold increase at 12 weeks of infection to a 6-fold decrease at 24 weeks. As *F*. *nucleatum* is able to aggregate and induce apoptosis in peripheral blood mononuclear cells [47], the decrease in FasL at 24 weeks of infection may be a physiological attempt to counter this cell death. Other serum cytokine changes in *F*. *nucleatum*-infected mice at 12 weeks indicated a strong systemic Th1 response, with significantly elevated levels of Th1-promoting IL-12 and CD30L. Strong Th2-response was evidenced by elevated levels of IL-4, correlating with the strong serum antibody response against the bacteria. Additionally, decreased levels of leukocyte chemoattractants relative to sham-infected mice at 12 weeks support a long-term response to *F*. *nucleatum* infection that leads to repressed inflammation. Both the Th2 response and inhibition of inflammation are maintained at 24 weeks of infection, although fewer cytokines overall are significantly altered, similar to the observed response to *P*. *gingivalis* infection [4], indicating a homeostatic balance is being re-established. In particular, the T cell chemoattractants ITAC, lymphotactin, MCSF and MIG, which are elevated in infected mice at 12 weeks, are no different from sham-infected mouse levels at 24 weeks.

When compared to *P*. *gingivalis* [4] and *T*. *denticola* [5] monoinfections, the changes we observed in aortic gene expression, serum cytokine levels, and aortic inflammatory cell counts suggest a potential mechanisms for the reduction of atherosclerotic plaque in 24-week *F*. *nucleatum*-infected mice involving Th2 driven immune responses and resolution of inflammation. *F*. *nucleatum*-induced gene expression changes at 24 weeks suggest a strong Th2 response, with more “immune response” genes affected than in either *P*. *gingivalis* or *T*. *denticola* monoinfections. As the aortic plaques detected in *P*. *gingivalis*-infected mice were smaller than those in the *T*. *denticola*-infected mice, an early Th2-driven response in the aorta may explain reduced plaque relative to *T*. *denticola*-infected mice. Additionally, a Th17-driven response in *P*. *gingivalis* and *T*. *denticola* monoinfections was detected by elevated levels of serum IL-17 at 12 weeks in *T*. *denticola*-infected mice and at 24 weeks in both *P*. *gingivalis* and *T*. *denticola*-infected mice that were not detected in *F*. *nucleatum*-infected mice may drive inflammation that promotes atherosclerotic lesion development.

Interestingly, the aortic expression of *serpinE1* and *serpinB2* in *F*. *nucleatum*-infected mouse aortas but not in *P*. *gingivalis*- or *T*. *denticola*-infected aortas [4,5] at 24 weeks, suggest a possible role of these serpins in resolution of inflammation. SerpinB2 is a plasminogen activator-inhibitor (also known as PAI-2) thought to play a role in reducing inflammation, although the exact part it plays is not yet known [48]. Expression of *serpinB2* is known to increase in monocytes in response to inflammatory cytokines including IFNγ, IL-1β and MCSF, as well as to bacterial LPS and lipoprotein fractions VLDL and LDL [48], and we detected elevated levels in *F*. *nucleatum*-infected mice while *T*. *denticola*-infected mice exhibited reduced expression and *P*. *gingivalis*-infected mice had no change in *serpinB2* expression. Tellingly, this protein is significantly elevated in gingival crevicular fluid from inflamed periodontal sites, where it is thought to protect against excessive tissue damage, and indeed SerpinB2 has been suggested to be the primary PAI in inflamed tissues [48]. Our data may suggest that *F*. *nucleatum* promotes anti-inflammatory responses in both gingivae and aortic tissues at 12 and 24 weeks, both by sustaining a Th2 response and repressing a Th1 response, and that elevated levels of *F*. *nucleatum* detected in PD-sites are a result of the organism taking advantage of a permissive environment, and are not due to *F*. *nucleatum*-driven inflammation.

SerpinE1, also known as PAI-1, is involved in resolution of inflammation, in particular by promoting macrophage migration away from sites of inflammation [49]. PAI-1-deficient C57BL/6 mice experience more severe Gram-negative bacterial pneumonia than transgenic mice overexpressing PAI-1, evidenced by greater bacterial growth and dissemination [50], which suggests that in this mouse strain SerpinE1 is important for clearing bacterial infection. In *F*. *nucleatum*-infected mice, *serpinE1* expression was elevated, which corresponded with the F4/80^+^ macrophage cell counts in aortic tissues at 24 weeks of infection. Fewer F4/80^+^ macrophages were detected in the intimal layer of 24-week-infected mice than 12-week-infected mice, indicating reduction of inflammation in the intimal layer, while greater numbers of F4/80^+^ macrophages were detected in the medial tissues than intimal tissues at 24 weeks. Together these observations suggest that the macrophages are migrating out of the aortic tissues towards the draining lymph nodes. The expression of *serpinE1* was not affected in *P*. *gingivalis*-infected mice, which experienced an increase in F4/80^+^ macrophages at 24 weeks relative to 12 weeks, but not among 24 week-infected and control mice. *T*. *denticola*-infected mice experienced elevated aortic expression of *serpinE1*, yet no aortic tissue F4/80^+^ staining observed. However, fibrinogen gene expression at 24 weeks in *T*. *denticola*-infected mice is less strongly down-regulated than in *F*. *nucleatum*- or *P*. *gingivalis*-infected mice, which may indicate greater fibrin deposition in *T*. *denticola*-infected aortic tissue, which may promote macrophage retention [5].

Several studies have examined the synergism between *F*. *nucleatum* and other bacterial pathogens *in vitro* and in rodent infection models [18–20,45,51], and have found that the inclusion of *F*. *nucleatum* results in greater pathogenic effects. In this regard and as suggested by our data, *F*. *nucleatum* may not act to promote atherogenesis alone, but may synergistically assist other organisms, particularly by disrupting cell-cell junctions, compromising the integrity of the endothelium, and enabling invasion [45]. For example, *F*. *nucleatum* may prime the host to infection by other organisms, as is observed with Influenza A facilitating pneumococcal infection by damaging tissues [52], compromising local immunity [52] and/or providing nutrition [53]. Despite the recorded ability of *F*. *nucleatum* to induce endothelial cell dysfunction [45], chronic oral *F*. *nucleatum* infection did not alter the levels of serum NO, which is a potent indicator of vascular endothelial cell dysfunction. It may be concluded from the observations in our study that due to the interdependence (physical, metabolic, and nutritional) of periodontal bacteria, no single periodontal bacterial species alone will effectively induce aortic disease pathology, rather it may be induced by a polymicrobial consortium.
